# Diagnostic significance of lung ultrasound in evaluation of heart failure: A cross-sectional study in comparison with clinical assessment, proBNP, chest X-ray, and echocardiography

**DOI:** 10.1097/MD.0000000000046722

**Published:** 2025-12-19

**Authors:** Ahmed Raed Alrubaye, Ahmed Dheyaa Al-Obaidi, Marafi Jammaa Ahmed, Aya Ahmed Shimal, Hashim Talib Hashim, Marwah Algodi, Abbas Al-Sharifi

**Affiliations:** a Internal Medicine Department, Imam Ali (Jawadr) Hospital, Baghdad, Iraq; b University of Baghdad, College of Medicine, Baghdad, Iraq; c Faculty of Medicine, Bahri University, Khartoum, Sudan; d College of Medicine, University of Baghdad, Baghdad, Iraq; e University of Warith Al-Anbiyaa, College of Medicine, Karbala, Iraq; f Department of Internal Medicine, Hackensack Meridian Health-Jersey Shore University Medical Center, Neptune City, NJ; g Department of Medicine, Al-Mustansiriyah College of Medicine, Baghdad, Iraq.

**Keywords:** acute heart failure, B-lines, diagnostic accuracy, ejection fraction, lung ultrasound, NT-proBNP, pulmonary congestion

## Abstract

Lung ultrasound (LUS) detection of B-lines is a useful bedside tool for evaluating pulmonary congestion in acute heart failure. This study aimed to assess the diagnostic performance of LUS compared to clinical assessment, N-terminal pro-B-type natriuretic peptide, chest X-ray, and echocardiography in patients with systolic heart failure. A cross-sectional study was conducted on 50 patients diagnosed with heart failure based on Framingham criteria and confirmed by echocardiography at Al Yarmook General Teaching Hospital, Baghdad (May–October 2021). LUS was performed across 16 lung zones to detect B-lines. Diagnostic validity was assessed using receiver operating characteristic curve analysis, referencing ejection fraction ≤ 30%, mitral inflow to annular velocity ratio (*E*/e*é*) ≥ 15, and N-terminal pro-B-type natriuretic peptide > 450 pg/mL. From the 16 lung zones, zone number 1 in the left side was with B lines findings in all (46) ultrasound positive patients, followed by zone number 1 in the right side were with positive B lines in (44) patients. Using ≥10 lung zones with B-lines as the diagnostic cutoff for acute heart failure, LUS demonstrated a sensitivity of 64.7% and specificity of 63.6% when ejection fraction ≤30% was used as the reference standard, and a sensitivity of 63.6% and specificity of 88.2% when *E*/*e′* ≥ 15 was used as the reference. While using pro-B-type natriuretic peptide > 450 pg/mL as reference for acute heart failure it showed that 4 lung zones with B lines will be the cutoff value with a sensitivity of 86.67% and specificity of 80.00%. Lung sonography could be considered a reliable tool for the assessment of pulmonary congestion in patients with acute heart failure. Using 10 lung zones with B-lines by ultrasound as a cutoff value for the diagnosis of acute heart failure. However, its accuracy may be limited by operator dependency and small sample size.

## 1. Introduction

Acute heart failure (AHF) is one of the leading causes of acute dyspnea in the emergency department (ED) and is associated with a higher risk of morbidity and mortality. In-hospital mortality is reported to be >10% and has remained stable in the last 30 years.^[1]^ As prognosis is associated with initiation time of specific therapies, current guidelines emphasize the importance of early diagnosis and treatment initiation to improve clinical outcomes.^[2]^

Despite impressive improvements in treatment strategies over the past 2 decades, heart failure (HF) morbidity and mortality remain substantially high worldwide. Pulmonary congestion, rather than low cardiac output, is considered the leading cause of hospital admissions and death among patients with HF. Physical examination is crucial for titrating medical treatment in these patients. Unfortunately, this traditional assessment has a good specificity but is not sensitive enough to detect elevated cardiac filling pressures.^[3]^ Recently, an increasing number of better diagnostic tools for AHF are available in the ED.^[4]^ However, there is likely room for improving the diagnostic approach to AHF from widely available routine tools, including chest radiography (chest X ray [CXR]). Chest radiography is a fast and inexpensive method performed systematically in the ED in patients with acute dyspnea.^[1]^ Different clinical congestion scores have been developed to evaluate signs of congestion in patients with HF.^[5–9]^ However, data comparing these scores with more established tools of decompensation are scarce. The aminoterminal portion of B-type natriuretic peptide (N-terminal pro-B-type natriuretic peptide [NT-proBNP]) is a powerful neuro-hormonal predictor of prognosis in HF and can be used to titrate therapy.^[5]^

Lung ultrasound (LUS) through B-line evaluation (formerly ultrasound lung comets) has been recently proposed as a simple, noninvasive, and semiquantitative tool to assess extravascular lung water.^[6,10]^ Recent bicentric data confirm the high diagnostic performance of pulmonary ultrasonography for acute heart failure, reinforcing its role as a frontline bedside test.^[11]^ B-lines have been shown to be correlated to NT-proBNP and *E*/*e′* levels in patients with acute dyspnea and after a stress test. LUS can also identify clinically silent pulmonary edema, suggesting that it could be added to the clinical evaluation to improve hemodynamic profiling and treatment optimization.^[7]^ This study was based on the assumption that LUS could reliably detect pulmonary congestion and provide diagnostic accuracy comparable to NT-proBNP, chest X-ray, and echocardiography in patients with acute heart failure. However, direct comparisons between LUS and other standard diagnostic tools in the context of acute decompensated heart failure, particularly in patients with systolic dysfunction, remain limited and inconsistent. This lack of comparative data restricts clinicians from fully integrating LUS into standard diagnostic algorithms. Therefore, this study addresses this gap by providing new evidence from Iraq, where such comparative data are scarce. The main objective was to evaluate the diagnostic performance of LUS in comparison with these standard diagnostic tools and to explore its role as a simple, rapid, and noninvasive bedside method for assessing decompensation in patients with systolic heart failure.

## 2. Methods and materials

### 2.1. Study design and setting

This cross-sectional study was conducted at Al Yarmook General Teaching Hospital in Baghdad, Iraq, over a 6-month period from May 1st to October 30th, 2021. The hospital’s emergency department (ER), internal medicine ward, and coronary care unit served as the recruitment sites for this study.

### 2.2. Study population

The study included 50 patients, who represented all eligible cases admitted during the study period. The study enrolled adult patients aged over 18 years who were admitted to the medical ER, internal medicine ward, or coronary care unit and met the diagnostic criteria for heart failure based on the Framingham classification.^[12]^ According to these criteria, a diagnosis of heart failure requires the presence of either 2 major criteria or one major plus 2 minor criteria. Major criteria included acute pulmonary edema, cardiomegaly, hepatojugular reflux, neck vein distension, paroxysmal nocturnal dyspnea or orthopnea, rales, third heart sound (S3), and elevated venous pressure. Minor criteria consisted of ankle edema, dyspnea on exertion, hepatomegaly, nocturnal cough, pleural effusion, tachycardia with a heart rate exceeding 120 beats per minute, and a reduction in vital capacity to less than two-thirds of the expected maximum. In addition, weight loss of >4.5 kg over 5 days in response to treatment was considered diagnostic as either a major or a minor criterion.

Patients were excluded if they had underlying chronic pulmonary conditions such as chronic obstructive pulmonary disease, interstitial lung disease, or pulmonary fibrosis; clinical or radiological suspicion of chest infections; or a history of bronchogenic carcinoma to minimize confounding, as these conditions can independently produce B-lines on LUS and mimic pulmonary congestion, potentially leading to misinterpretation of results. All exclusions were confirmed with chest computed tomography. Pulmonary function tests were not performed because the pulmonary function laboratory was closed due to restrictions during the COVID-19 pandemic.

### 2.3. Data collection and procedures

Data collection was conducted over 3 months, approximately 3 days per week, through direct patient interviews and clinical assessments carried out by the investigator using a structured questionnaire. This questionnaire was reviewed by 2 senior cardiologists to ensure content validity, clarity, and consistency before data collection. This instrument collected sociodemographic information such as age, sex, smoking status, and comorbidities, in addition to documentation of clinical findings based on Framingham criteria.

Patients who met the Framingham criteria underwent a transthoracic echocardiographic examination using a GE SN70 machine equipped with a phased array probe. Those with a reduced ejection fraction (EF) were further evaluated and included in the study. Additional echocardiographic parameters, including the *E*/*e′* ratio and diastolic function, were assessed following the most recent recommendations of the American Society of Echocardiography.

Blood samples were obtained for NT-proBNP testing using a qualitative rapid assay. A single drop of whole blood and 2 drops of buffer were applied to the cassette, with results interpreted after 10 minutes. A positive result corresponded to a serum NT-proBNP level exceeding 450 pg/mL, as defined by the manufacturer’s specifications.^[13,14]^

Chest radiographs were obtained in the posteroanterior view, with the patient in a standing position and full inspiration. All radiographs were interpreted by a radiologist and assessed for signs of pulmonary congestion, including pulmonary vein cephalization, peribronchial cuffing, perihilar haze, Kerley B-lines, batwing pattern, cardiothoracic ratio, and pleural effusion.

LUS was also performed using the GE SN70 machine with the same phased array probe. Patients were examined in a semi-sitting position, and the presence of B-lines was assessed using a standardized 16-zone protocol, which included 8 regions per lung (anterior, lateral, and posterior areas).^[15]^ A positive LUS result was defined as the presence of 3 or more B-lines in at least 2 lung zones on each side.

### 2.4. Statistical analysis

All data were entered into Microsoft Excel 2019 and analyzed using SPSS software version 24 (IBM Corp., Armonk). Continuous variables were presented as means and standard deviations or as medians with interquartile ranges, depending on data distribution. Categorical variables were expressed as frequencies and percentages. Missing data were minimal and handled by case-wise deletion; only complete datasets were included in the final analysis to maintain accuracy. Group comparisons were performed using the Chi-square test for categorical data and the independent-sample *t* test for continuous data, as appropriate. The diagnostic accuracy of LUS in detecting decompensated heart failure was evaluated using receiver operating characteristic (ROC) curves. The optimal cutoff point for LUS findings was identified as the point on the curve that maximized both sensitivity and specificity. All findings were summarized in tables and figures.

### 2.5. Ethical considerations

Ethical approval for the study was obtained from the Arabic Board of Medical Specializations. All procedures were performed in accordance with the principles outlined in the Declaration of Helsinki.

## 3. Results

In this study, 50 patients diagnosed to have acute heart failure by Framingham criteria and then by echocardiography were included. There were 42 (84%) of patients >40 years old. The male-to-female ratio was (9.6: 7). Nearly half had hypertension, two-thirds had diabetes mellitus, and about 3-quarters had coronary artery disease. Dyslipidemia and smoking were less frequent. All the patients were with diastolic dysfunction and the majority of patients 23 (46%) were with grade II, and 23 (46%) grade III, mean age of the included patients was (58.22 ± 14.11), the patients were within mean EF (35.12 ± 6.44), and mean *E*/*e′* was (20 ± 7), as illustrated in Table 1.

**Table 1:** Demographic characteristics of the included participants.

Demographic characteristics of the study sample (n = 50)					
Variables		N.		%	
Age groups (in years)	≤40	8		16.0	
	>40	42		84.0	
Sex	Male	29		58.0	
	Female	21		42.0	
Hypertension	Yes	24		48.0	
	No	26		52.0	
Diabetes mellitus	Yes	30		60.0	
	No	20		40.0	
Coronary artery diseases	Yes	37		74.0	
	No	13		26.0	
Dyslipidemia	Yes	8		16.0	
	No	42		84.0	
Smoking	Yes	20		40.0	
	No	30		60.0	
LV diastolic dysfunction	Grade I	4		8.0	
	Grade II	23		46.0	
	Grade III	23		46.0	

Variables	Mean	SD	Range	Maximum	Minimum
Age (in yr)	58.22	14.11	53	82	29
Ejection fraction %	35.12	6.44	26	48	22
*E*/*e′*	20	7	30	40	10

LV = left ventricle of the heart.

**Figure 1.**
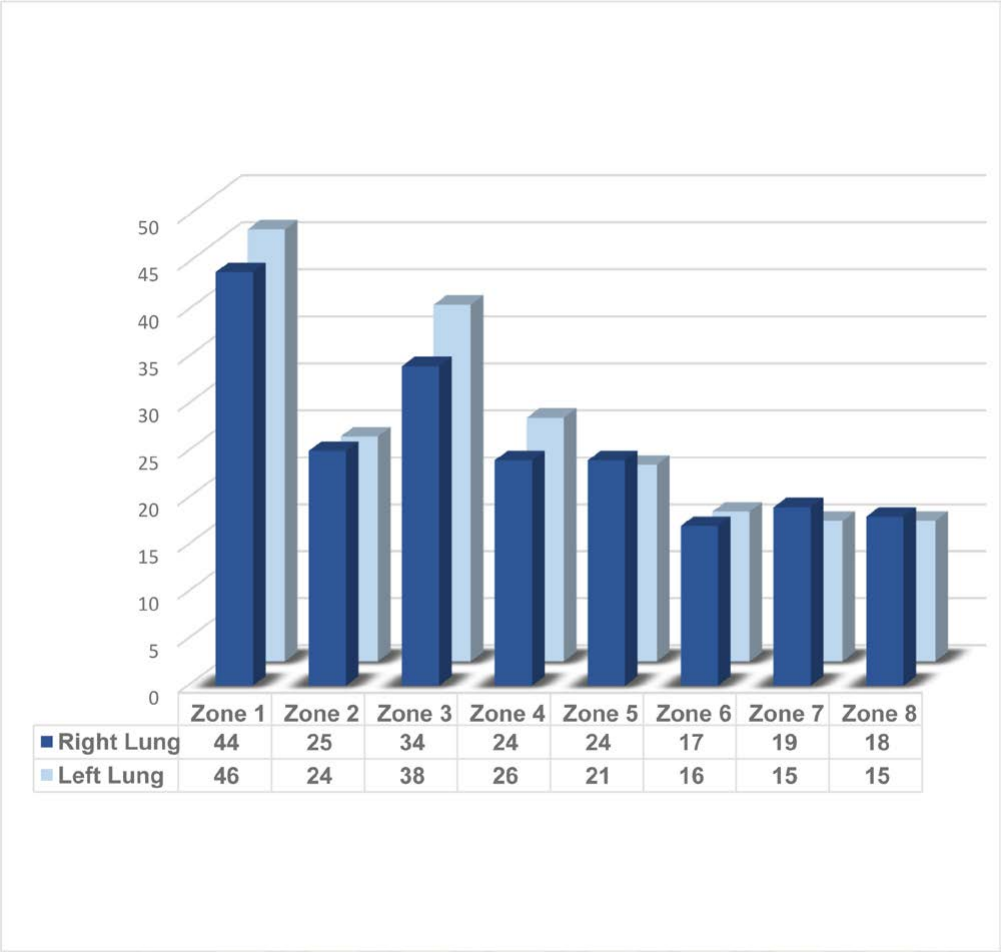
Frequency of lung zone with B-lines by ultrasound among study patients (n = 50).

For LUS, a 16-zone method was used by dividing the chest into 8 zones for each lung (right and left). Figure 1 shows of the 16 lung zones, zone number 1 on the left side was associated with B-line findings in all 46 ultrasound-positive patients, followed by zone number 1 on the right side was associated with positive B lines in 44 patients.

### 3.1. Optimal cutoff value

We compare the number of zones with B-lines by LUS (range 0–16) in the 50 patients with heart failure with *E*/*e′* ≥ 15 as reference for decompensation, EF ≤ 30 as reference for severe heart failure, and with pro-B-type natriuretic peptide (proBNP) ≥ 450 pg/mL as reference for decompensated heart failure.

### 3.2. Ejection fraction ≤ 30 as reference

**Figure 2.**
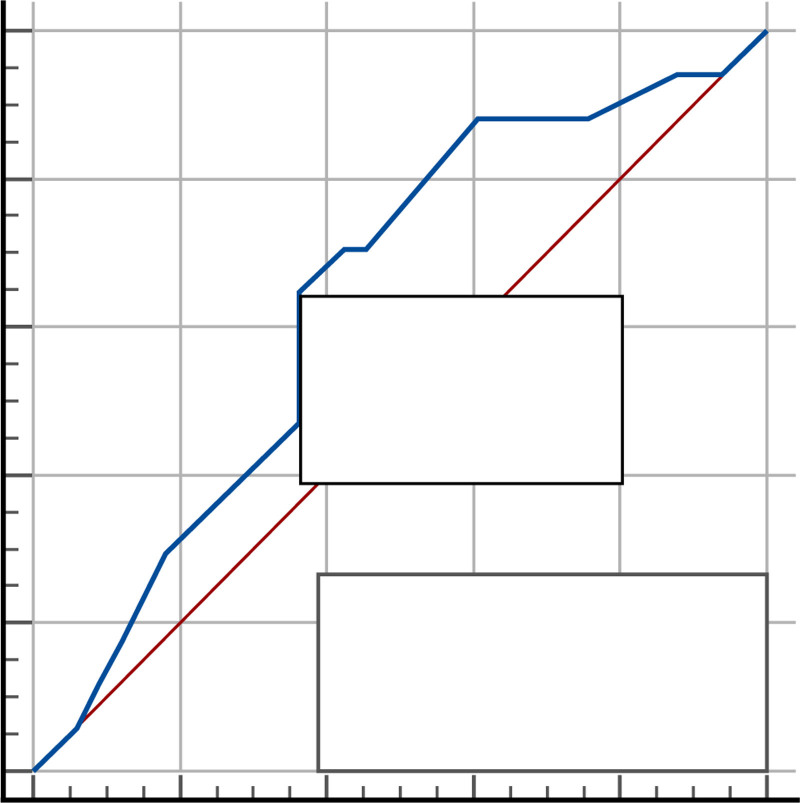
ROC curve for lung ultrasound with ejection fraction ≤ 30 as reference for severe heart failure. ROC = receiver operating characteristic.

The ROC curve for LUS in comparison to EF ≤ 30 showed an area under the curve (AUC) of 63.5%, with moderate sensitivity and specificity at a cutoff of >10 positive lung zones. See Tables 2 and 3, and Figure 2.

**Table 2:** ROC curve results for lung ultrasound using ejection.

Cut off value	AUC %	95% CI	*P*-value
>10 zones	63.5%	48.7 −76.7%	.099

95% CI = 95% confidence interval, AUC = area under curve, ROC = receiver operating characteristic.

**Table 3:** Validity of screening tests for lung ultrasound using ejection.

Sensitivity %	Specificity %	PPV%	NPV%	Accuracy %
64.71%	63.64%	47.8%	77.8%	28.34%

95% CI = 95% confidence interval, AUC = area under curve, NPV% = negative predictive value%, PPV% = positive predictive value%.

### 3.3. *E*/*e′ *≥ 15 as reference

**Figure 3.**
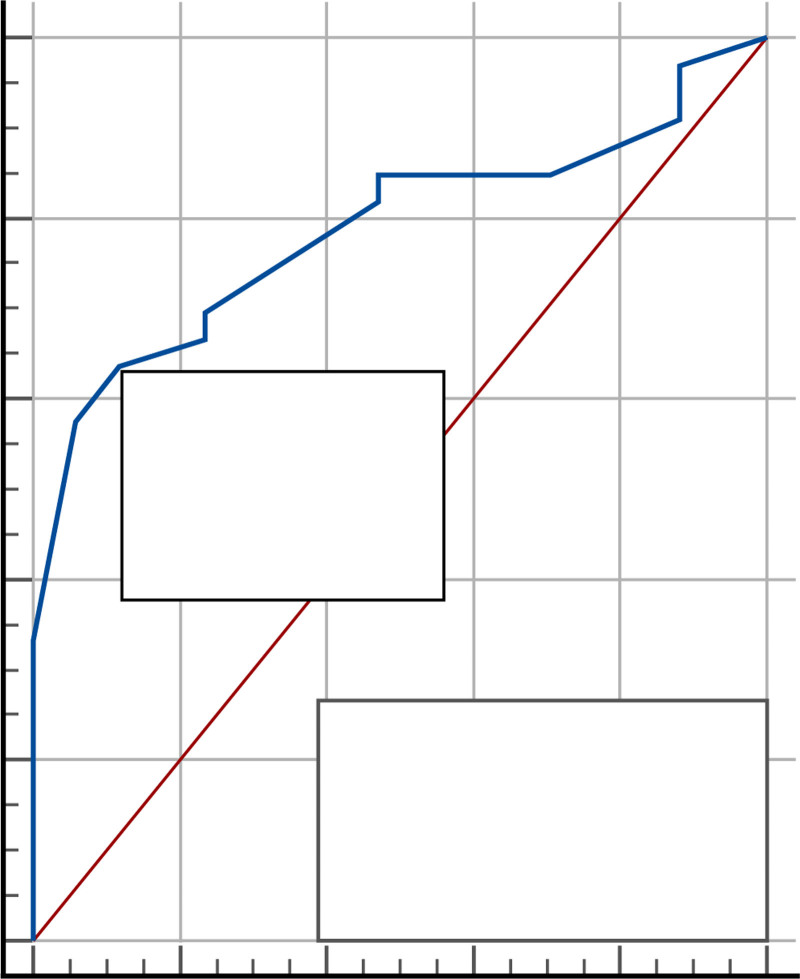
ROC curve for lung ultrasound with *E*/*e′* ≥ 15 as reference for decompensated heart failure. ROC = receiver operating characteristic.

ROC curve for LUS in comparison to *E*/*e′* ≥ 15 yielded an AUC of 78.8% at >10 zones as a cutoff value, providing a sensitivity of 63.6%, and specificity of 88.2%. As shown in Tables 4 and 5, and Figure 3.

**Table 4:** ROC (receiver operating characteristic) curve of lung ultrasound using *E*/*e′* ≥ 15 as reference.

Cut off value	AUC %	95% CI	*P*-value
>10 zones	78.8%	64.9−89.1%	<.001

95% CI = 95% confidence interval, AUC = area under curve.

**Table 5:** Validity of lung ultrasound using *E*/*e′* (early mitral inflow/annular velocity) ≥ 15 as reference.

Sensitivity %	Specificity %	PPV%	NPV%	Accuracy %
63.64%	88.24%	91.3%	55.6%	78.8%

95% CI = 95% confidence interval, AUC = area under curve, NPV% = negative predictive value%, PPV% = positive predictive value%.

### 3.4. ProBNP > 450 pg/mL as reference

**Figure 4.**
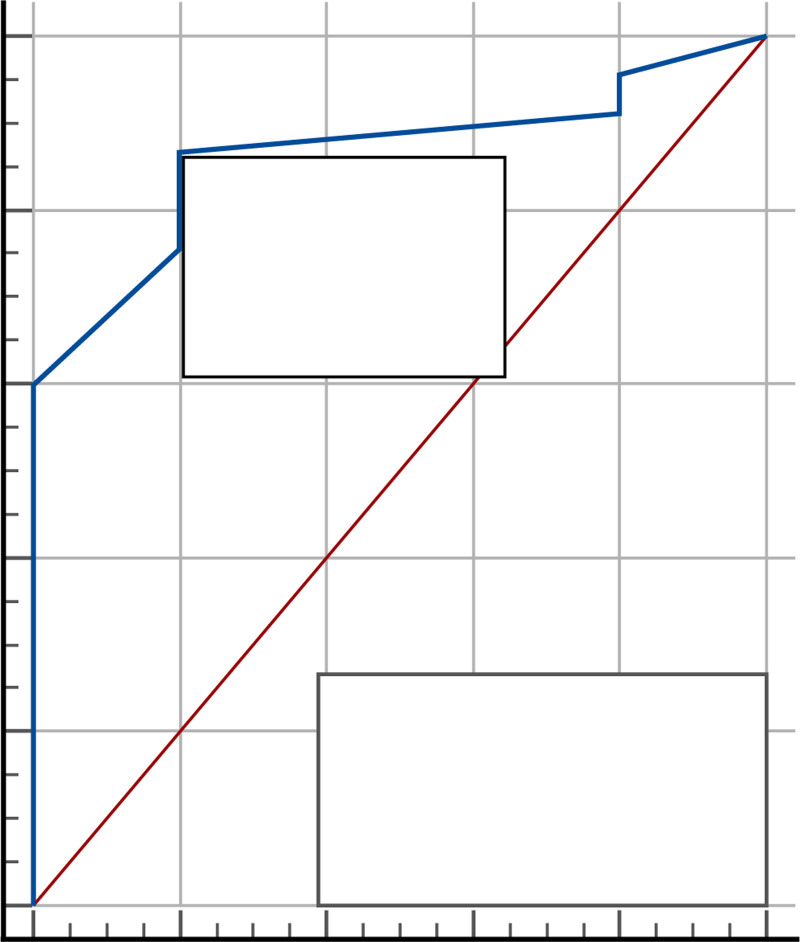
ROC curve for lung ultrasound with proBNP > 450 pg/mL as reference for decompensated heart failure. ProBNP = pro-B-type natriuretic peptide, ROC = receiver operating characteristic.

When NT-proBNP > 450 pg/mL was used as the reference, LUS demonstrated the highest diagnostic accuracy, with an AUC of 86.4% and balanced sensitivity and specificity at a cutoff of >4 lung zones. When NT-proBNP > 450 pg/mL was used as the reference, LUS demonstrated the highest diagnostic accuracy, with an AUC of 86.4% and balanced sensitivity and specificity at a cutoff of >4 lung zones. As shown in Tables 6 and 7 and Figure 4.

**Table 6:** ROC curve of lung ultrasound using BNP (B-type natriuretic peptide) > 450 pg/mL as reference.

Cut off value	AUC %	95% CI	*P*-value
>4 zones	86.4%	73.8−94.5%	< 0.001

95% CI = 95% confidence interval, AUC = area under curve, ROC = receiver operating characteristic.

**Table 7:** Validity of screening tests for lung ultrasound using B-type natriuretic peptide (BNP) > 450 pg/mL as reference.

Sensitivity %	Specificity %	PPV %	NPV %	Accuracy %
86.67%	80.00%	97.5%	40.0%	66.67%

95% CI = 95% confidence interval, AUC = area under curve, NPV% = negative predictive value%, PPV% = positive predictive value%.

The number of lung zones with B lines in ultrasound was significantly associated with proBNP level, where the highest proportion of patients with ≥10 zones in LUS 75.6% (34) were with proBNP level > 450 pg/mL, and the highest proportion of patients with <10 zones in LUS 80% (4) were with ≤450 pg/mL (*P* = .010).

From the total 17 patients with EF ≤ 30, 15 (88.2%) of them were with ≥10 zone in LUS with B lines, while from the total patients with EF > 30, 20 (60.6%) were with <10 zones in LUS and this difference was significant (*P* = .043).

Patients with an *E*/*e′* ratio ≥ 15 were significantly with a higher frequency of 27 (81.8%) for having ≥10 zones by LUS (*P* = .011).

Severity of diastolic dysfunction was significantly associated with the number of zones with B-lines by LUS (*P* = .019), whereas the highest proportion of patients with grade II and grade III were with ≥10 zones (60.9% [14], 87% [20] respectively).

By chest X-ray a total of 34 patients showed signs of compensation, from them 82.4% (28) patient were with ≥10 zones with B-lines by LUS, whereas 56.3% of (9) of the patients who were with normal chest X-ray were with <10 zones with B-lines by LUS and this association was significant (*P* = .005). All clarified in Table 8.

**Table 8:** Number of zones with B lines by lung ultrasound in association with other parameters of heart failure (n = 50).

Variables		Total	Frequency of Lung zones with B lines by US				*P*-value
			<10		≥10		
			N.	%	N.	%	
ProBNP	>450 pg/mL	45	11	24.4	34	75.6	.010*
	≤450 pg/mL	5	4	80.0	1	20.0	
Ejection fraction	≤30	17	2	11.8	15	88.2	.043*
	>30	33	20	60.6	13	39.4	
*E*/*e′*	<15	17	9	52.9	8	47.1	.011*
	≥15	33	6	18.2	27	81.8	
Grading of diastolic dysfunction	Grade I	4	3	75.0	1	25.0	.019*
	Grade II	23	9	39.1	14	60.9	
	Grade III	23	3	13.0	20	87.0	
Chest X-ray	Compensated	34	6	17.6	28	82.4	.005*
	No compensation	16	9	56.3	7	43.8	

ProBNP = pro-B-type natriuretic peptide.

* Chi-square is significant at *P* = .05.

## 4. Discussion

LUS had been increasingly used in intensive care unit settings over the last 2 decades to evaluate patients with acute heart failure. Physicians must integrate several measurements to assess pulmonary congestion, as there is no individual parameter that reliably detects congestion.^[16]^ Guidelines and recent reviews recommend combining cardiac and LUS to assess congestion comprehensively in acute heart failure.^[17]^ Novel updated methods in cardiac imaging and clinical chemistry make it possible to detect failure at an early stage. LUS is particularly helpful in assessing congestion, and it has demonstrated diagnostic, and prognostic value in acute heart failure as it is relatively easy to learn and give a quick assessment of the presence of pulmonary congestion.^[18]^ Contemporary studies further corroborate that LUS reliably detects pulmonary congestion and can perform comparably to conventional imaging in the ED setting.^[11]^ This study is to investigate lung sonography performance in assessing decompensation in patients with systolic heart failure.

The sociodemographic features and associated comorbidities in our study sample are slightly differs from a prevalence study investigated the associations of chronic conditions with heart failure done in Minnesota/USA in 2020 in which 2643 matched pairs was involved, with mean age 76.2 years and less than half of them were men (45.6%), the studies patients with heart failure have a high prevalence of many associated chronic conditions like arrhythmia, hypertension, and coronary artery disease and DM, respectively; most of those heart failure patients were smokers.^[19]^ These comorbidities, particularly hypertension, diabetes mellitus, and coronary artery disease, may act as confounders, as they independently contribute to changes in cardiac filling pressures and pulmonary congestion, potentially influencing both NT-proBNP levels and B-line formation on LUS.

This is also agreed with Owei et al in 2016 who underline the need to include a multi-morbid cases in the heart failure studies to create a broader comprehensive image, closer to real-life populations. These combined pathologies bear the potential to increase the liability for acute presentations and decompensation of heart failure. Consequently, this comorbidity burden leads to more frequent complications and rehospitalization.^[20]^

All the patients in this study had diastolic dysfunction, this is consistent with a study that recruited 91 patients with heart failure in Bucharest, 2019, in which a large percentage of the patients had poor EF and grade II heart failure. The *E*/*e′* ratio mean value for the cases on admission was 14.51 ± 4.61. Which is less than our results.^[21]^

According to LUS, this study revealed that B-line findings were positive in the left side of 46 patients, and they were positive in 44 patients on the right side. LUS in comparison to EF ≤ 30 yielded a sensitivity of 64.71% and specificity of 63.64%. while LUS in comparison to *E*/*e′* ≥ 15 yielded a sensitivity of 63.6% and specificity of 88.2%.While for LUS in comparison to proBNP > 450 pg/mL, the sensitivity was 86.67%, and the specificity of 80.00%.This is in disagreement with Platz et al study, who used a simplified 4-zone songraphic method in patients with AHF who later on improved with therapy. Among 349 patients with a mean EF 39%, the sum of B-lines in 4 zones ranged from 0 to 18. The *E*/*e′* ratio threshold value of 13.80 was identified (sensitivity: 78.60%, specificity: 55%). For proBNP, the sensitivity was 86 %, and the specificity of 79%.^[22]^ Different scanning protocols (4-, 8-, and 16-zone) yield different B-line counts and diagnostic cutoffs. For example, an 8-point method used for ED monitoring showed good diagnostic performance and 72-hour responsiveness.^[23]^

Another prospective cohort study by Lee et al involved patients with clinical suspicion of heart failure who underwent echocardiography, LUS, and proBNP measurement during their first visit to the cardiology outpatient clinic. The study found that the predictive value of B-lines in LUS is almost similar to that of NT-proBNP. They also found that more than 15 B-lines in LUS was associated with a significantly worse outcome.^[24]^

Many studies performed with pediatric populations show that, in an appropriate clinical context, the presence of diffused B-lines in LUS in some cases might be compatible with a multifocal infection.^[25]^ LUS is cheap, feasible, portable, and safe. Clinicians are working hard to master the best way to use this new tool, but the tool has some limitations. When predicting pulmonary edema in those patients, the B line appears to have reached a ceiling, trading off between sensitivity and specificity. In our study, we obtained acceptable Specificity values but lower Sensitivity values, as 36.4% of the patients had LUS findings with pathological B-lines. We believe that this can be explained by the fact that the diagnosis could have possibly a respiratory infection that triggered such decompensation and was not viewed in routine diagnostic chest X-rays. In addition, undetected mild interstitial or postinfectious lung changes could have contributed to false-positive B-lines, reducing specificity. Although the interpretation of LUS images requires training and specific experience, ultrasonographic signs are well categorized and operator dependence is minimal; nevertheless, the high interobserver agreement should be obtained.^[26]^

In the present study, the number of lung zones with ≥10 B lines in LUS was significantly associated with EF > 30, *E*/*e′* ratio ≥ 15, proBNP level > 450 pg/mL, severity of diastolic dysfunction, and abnormal chest X-ray findings.

This is in comparison with a recent meta-analysis study which stated that LUS is more sensitive and accurate than CXR in diagnosis of pulmonary edema in acute heart failure, showing higher sensitivity for LUS: 0.88 versus CXR: 0.73. Consistent with Maw et al’s systematic review and meta-analysis, LUS shows higher sensitivity than chest radiography for identifying cardiogenic pulmonary edema.^[10]^ Although no difference in specificity between the 2, it also stated that the presence of 1 or 2 B-lines could be normal, but the presence of ≥3 B-lines is usually consistent with pulmonary congestion and heart failure, representing the chest X-rays equivalent of Kerley B lines. That’s why, and in the right clinical setting, the presence of ≥3 B-lines is highly sensitive and specific for pulmonary edema irrespective of operator expertise.^[10,27]^

While these findings support the growing evidence for LUS as a practical bedside tool, the results should be interpreted within the context of the study’s methodological limitations. Besides its strengths, several limitations should be acknowledged. Despite the relatively small sample size and the difficulties in obtaining enough images in LUS image interpretation, this study is important for several reasons. First, the addition of LUS to clinical assessment of those ill patients provides invaluable actionable information that could be used to assess prognosis. Second: the study contributes to the emerging scientific literature showing the value of LUS in the cardiac intensive care setting which is to the extent to our knowledge the first in our country in this setting.

Future large-scale, multicenter studies are strongly recommended to validate the proposed diagnostic cutoffs and to clarify the incremental value of LUS when combined with echocardiography and natriuretic peptide testing. Because there is no gold standard test for the diagnosis of acute heart failure, we recommend the use of LUS in evaluating acute heart failure, especially when more than 10 lung zones show B-lines.

## 5. Conclusion

According to the findings of this study, lung ultrasonography appears to be a promising and practical adjunctive tool for assessing pulmonary congestion in patients with acute heart failure. It may provide additional diagnostic value when used alongside echocardiography, natriuretic peptide testing, and clinical assessment. However, the findings of this study should be interpreted with caution due to the small sample size and single-center design. Further large-scale, multicenter studies are recommended to validate these results and to establish standardized diagnostic cutoffs for the use of LUS in heart failure evaluation.

## Author contributions

**Conceptualization:** Ahmed Raed Alrubaye, Abbas Al-Sharifi.

**Data curation:** Ahmed Dheyaa Al-Obaidi, Hashim Talib Hashim.

**Formal analysis:** Marwah Algodi.

**Investigation:** Aya Ahmed Shimal.

**Methodology:** Marafi Jammaa Ahmed, Aya Ahmed Shimal, Hashim Talib Hashim.

**Supervision:** Ahmed Raed Alrubaye, Abbas Al-Sharifi.

**Validation:** Ahmed Dheyaa Al-Obaidi.

**Visualization:** Marwah Algodi.

**Writing – original draft:** Ahmed Dheyaa Al-Obaidi, Marafi Jammaa Ahmed, Aya Ahmed Shimal, Hashim Talib Hashim, Marwah Algodi.

**Writing – review & editing:** Ahmed Raed Alrubaye, Marafi Jammaa Ahmed, Abbas Al-Sharifi.
